# Racism as context and resilience as infrastructure: the racism–resilience response framework for equity-focused implementation

**DOI:** 10.3389/frhs.2026.1955240

**Published:** 2026-10-13

**Authors:** Tiffany J. Grant

**Affiliations:** Research & Data Services, University of Cincinnati Libraries, Cincinnati, OH, United States

**Keywords:** community resilience, health equity, implementation determinant, implementation science, medical mistrust, structural racism

## Abstract

Past and present experiences of racism in minoritized communities shape the environments where health interventions are implemented and create a distinct health infrastructure built from adaptive resilience responses. Implementation science has a unique opportunity to advance health equity by examining the barriers and facilitators that determine implementation success. Yet most implementation frameworks overlook racism mediated resilience, treating racism as a background risk factor rather than a multilevel implementation determinant. They also tend to imply a linear model of implementation, though interventions are introduced into dynamic systems shaped by structural racism, institutional disinvestment, organizational constraint, mistrust, and community-generated resilience. In these settings, implementation is an iterative process shaped by interacting forces across levels and over time. This article brings together evidence on how racism drives health inequities in minoritized communities and on the adaptive strategies communities use to navigate these conditions—such as mutual aid networks, community-based organizations, faith institutions, and trusted messengers. I argue that these resilience systems function as parallel public health infrastructures and that implementation strategies that fail to engage them miss opportunities to increase implementation success and promote health equity. Building on the Health Equity Implementation Framework, Public Health 3.0, and Ellis's Community Resilience model, I propose the Racism–Resilience Response Framework (R3F). This equity-centered implementation framework positions racism and community resilience as multilevel implementation determinants and conceptualizes implementation as a systems-oriented, iterative process rather than a linear one. The R3F outlines a four-step participatory process: 1.) Team formation and framing, 2.) Multilevel assessment and mapping, 3.) Determinant mapping and strategy design, and 4.) Equity-focused evaluation that embeds multisector collaboration and community-generated resilience strategies into implementation planning and delivery. Together, these steps embed multisector collaboration and community-generated resilience strategies into implementation planning and delivery. Although the framework requires empirical testing, it offers implementation scientists a practical approach for confronting racism directly and advancing more equitable, community-grounded implementation practice.

## 1 Introduction

Modern health research has generated a growing body of evidence-based interventions (EBIs) across clinical care, public health, and community settings. Although knowledge continues to grow, a major challenge is translating available EBIs into successful practice across these settings. This “knowledge-practice gap” can delay implementation by up to 17 years, and only 14% of those interventions are successfully implemented in the target population (1). Implementation science emerged to address this gap by examining how to deliver effective interventions in real-world contexts, identifying implementation barriers and facilitators, improving processes, and ensuring that evidence-based practices are adopted, adapted, and sustained. Because it focuses on issues that impact uptake and sustainability, implementation science is well suited to address health disparities and close the disparity gap observed in minoritized communities. However, implementation strategies that have health equity as a goal have only recently begun to emerge. To achieve equitable outcomes, implementation science must explicitly identify structural racism as both a driver of health disparities and as a determinant involved in unsuccessful implementation efforts (2, 3).

Implementation science recognizes that interventions are never introduced in neutral environments, but rather within complex, multilevel contexts that shape how they are delivered, experienced, and sustained in real-world settings. Although “context” has no single standard definition, Squires suggests that a core set of context domains relevant to healthcare likely exists. Failure to consider context limits the generalizability of study findings across settings, contributes to differences in outcomes measured across studies, and ultimately shapes whether implementation efforts succeed or fail (4, 5). In practice, context must be understood not only in organizational or systems terms, but also in relation to the everyday lives of patients and communities. Context is created by the historical, social, and political conditions in which people live and seek care, and health-seeking behaviors are inseparable from these conditions (6). This is especially true in minoritized communities, where encounters of racism shape health-seeking behaviors and determine how individuals engage with the healthcare system.

Racism operates at structural, institutional, and interpersonal levels to unjustly disadvantage people from minoritized groups and unfairly benefit people from White ‘majority’ groups (7). It is a fundamental driver of health inequities, creating disparities that result in higher rates of premature death, chronic illness, and overall poorer health outcomes (8, 9). These disparities are rooted in, and reinforced by, a long history of explicit medical racism, including abuses such as the Tuskegee Study of Untreated Syphilis in the Negro Male, the non-consensual use of Henrietta Lacks' cells, and numerous other exploitative experiments involving Black and other marginalized communities (10). This historical trauma is compounded by contemporary physician implicit bias and is further exacerbated by American medical training that has historically conflated race with biology and embeds racist assumptions into medical practice (10). Together, these experiences foster deep mistrust in the medical system and significantly alter how people engage with it. EBIs are implemented in communities whose histories of racism, mistrust, and resilience shape how new practices are received. To close the knowledge–practice gap and deliver equitable outcomes in minoritized communities, implementation strategies must account for these adaptive responses to racism.

This work addresses this gap by explicitly identifying racism as an implementation barrier, foregrounding historical resilience strategies as a resource for improving health outcomes across clinical, organizational, and community settings, and conceptualizing racism and resilience as interacting systems-level determinants that require iterative, multilevel implementation strategies. The Racism–Resilience Response Framework (R3F) is designed to guide implementation planning at both the individual and population levels. At the individual level, R3F can inform clinical interventions by identifying how racism, mistrust, and resilience shape patient engagement, care delivery, and provider–patient interactions. At the population level, it can guide community and public health interventions by identifying how structural racism, institutional disinvestment, and community-generated resilience systems shape reach, uptake, and sustainability. Building on current health equity implementation frameworks and drawing from public health frameworks such as Public Health 3.0 and the Community Resilience Model, R3F underscores multisector collaboration, structural and social determinants, and community-defined resilience. It shows how adaptive, community-based responses to structural racism can be leveraged to mitigate these disparities. In this work, I synthesize evidence on racism and community resilience as multilevel implementation determinants, describe the framework's components, and outline its application to implementation planning in minoritized communities.

## 2 Racism as a multilevel implementation determinant

Structural racism “refers to the processes of racism that are embedded in laws (local, state, and federal), policies, and practices of society and its institutions that provide advantages to racial groups deemed as superior, while differentially oppressing, disadvantaging, or otherwise neglecting racial groups viewed as inferior” (11). Public health research often and incorrectly uses race as a proxy for racism despite race having neither a biological origin nor inherent health effects. Although much research focuses on psychosocial impacts and bias in healthcare settings (2) racism is much more pervasive, operating far more broadly through enduring US policies and practices that have harmed minoritized groups.

Federal policies enforcing racial residential segregation have been a major structural driver of health disparities in Black communities (12–14). Contemporary racial segregation is a direct result of redlining practices institutionalized during the Jim Crow era. Redlining was the lawful, systematic withholding of low-interest home mortgages across different neighborhoods. Redlined areas were predominantly inhabited by Black residents who faced these discriminatory practices that limited their ability to purchase homes elsewhere, thus perpetuating racial segregation (15). By codifying racial exclusion in housing, redlining concentrated poverty in Black communities through limited resource allocation, educational access, and employment opportunities (11, 16). A 2023 study of 213 counties in 37 states found that redlined areas are still disproportionately inhabited by racial minorities, and they rank consistently worse across all Social Determinants of Health (SDoH) domains (15).

An individual's neighborhood is a critical determinant of education, employment, socioeconomic status (SES), and ultimately health. An earlier study found that a one standard deviation reduction in segregation eliminates approximately one-third of the difference between Black individuals and White individuals in most outcomes (17). Residents of racially segregated neighborhoods are more likely to have lower SES, which is strongly correlated with poor health outcomes (16). Racism has been identified as a SDoH because it structures the distribution of money, power, and resources, driving inequities in housing, income, education, and ultimately health (18). These inequities contribute to higher rates of chronic disease, limited access to care, delayed treatment, and increased premature mortality. A 2018 study linked racial segregation to lower survival rates among Black Americans aged 35 to 75, and found that poverty, discrimination, and housing disparities were contributing factors to this survival gap (19). The authors concluded that eliminating racial segregation and equalizing the socioeconomic status of Black and White Americans would essentially eliminate this survival gap.

Not only do racism and segregation shape risk exposure, but they also play critical roles in the design, distribution, and function of the health care infrastructure in minority communities. Historically redlined and segregated neighborhoods are more likely to experience hospital closures, fewer primary care providers and pharmacies, and longer travel times to high-quality facilities, creating structural barriers to quality healthcare (16, 20, 21). Even when healthcare is available, minoritized patients are more likely to experience discrimination and bias, including longer wait times, inadequate pain treatment, lower rates of transplants, and microaggressions that result in underuse of services (18).

Shelton argues that “implementation science has an explicit opportunity to advance health equity by including structural racism as part of the broader context in which health inequities are embedded, and to consider it a determinant of equitable implementation of evidence-based interventions and implementation strategies” (2). Facilitating health equity requires recognizing racism not as a background risk factor but a core multilevel implementation determinant operating across levels of context. These contextual effects affect the implementation of clinical services delivered to individual patients, as well as the reach, adoption, and sustainability of community and population-level interventions.

At the structural level, racist housing, education, and policies determine whether communities have access to stable healthcare facilities, transportation, and insurance coverage, and thus affect the feasibility and reach of EBIs. In communities lacking stable institutions, infrastructure, or the conditions necessary to provide supportive care, even well-designed interventions may face reduced uptake and limited sustainability (22). At the organizational level, racist norms and policies influence the availability of resources, clinic location, referral patterns, and institutional accountability for equity, leaving organizations that serve minoritized communities less resourced and more constrained in their ability to implement EBIs successfully. At the interpersonal and individual level, racism manifests through discriminatory interactions between provider and patient, stereotype- and bias-driven decision-making, and internal mistrust within relationships. These types of racism affect how patients, families, and providers experience, interpret, and interact with new interventions, and they impact uptake and implementation success.

Together, these interrelated levels demonstrate how racism structures the implementation landscape by shaping the facilitators and barriers that determine whether interventions achieve equitable impact. Because implementation processes occur within the same multilevel systems through which racism operates (Figure 1), racism must be considered a cross-cutting determinant of implementation context and strategy effectiveness.

**Figure 1:**
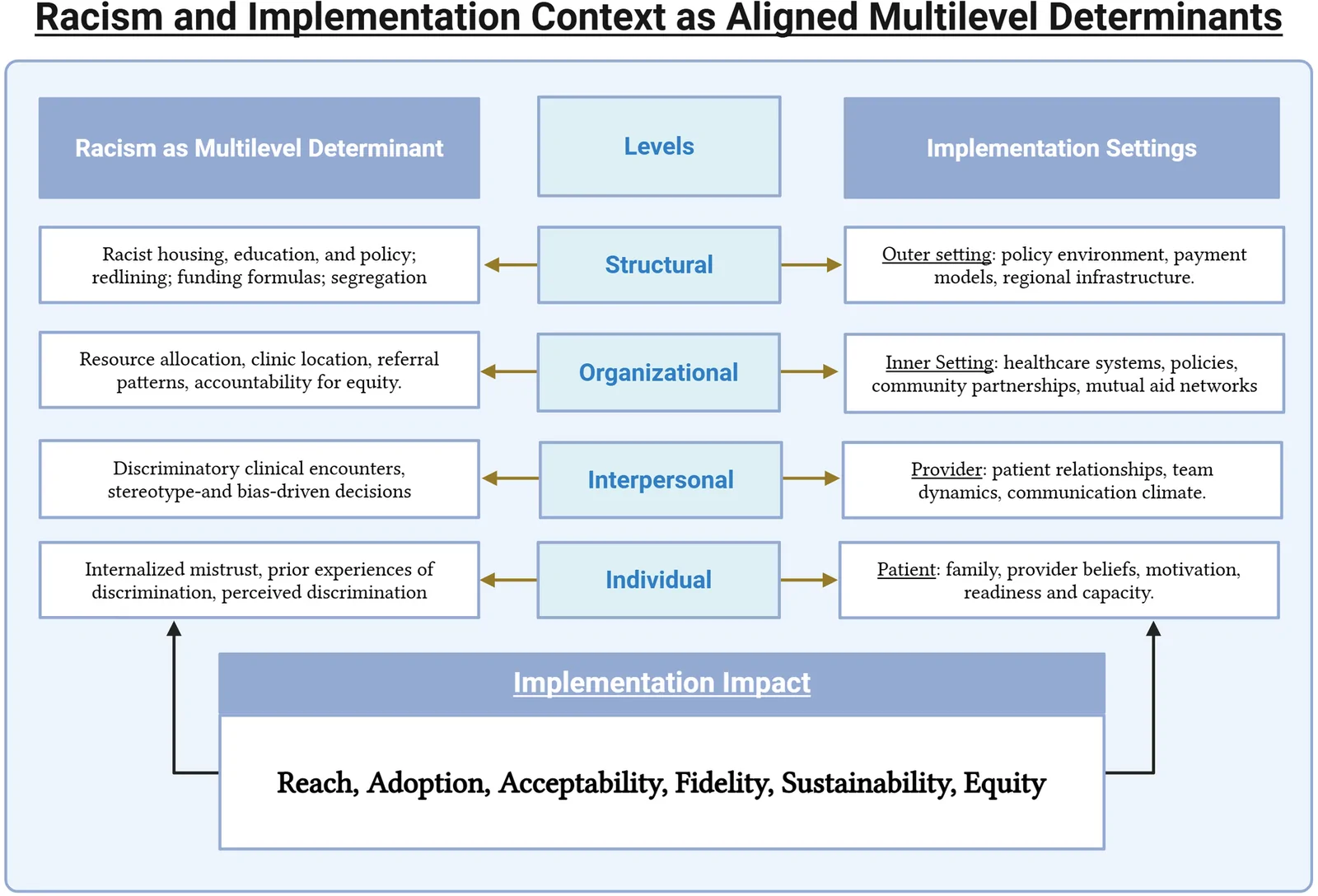
(23)**.** Racism and implementation context as aligned multilevel determinants. The diagram illustrates how racism operates as a multilevel determinant across structural, organizational, interpersonal, and individual levels, and how these levels align with common implementation settings (outer setting, inner context, provider, and patient). At each level, specific manifestations of racism (e.g., redlining, resource allocation, discriminatory clinical encounters, internalized mistrust) correspond to contextual features of implementation settings (e.g., policy environment, clinic characteristics, team dynamics, patient beliefs and readiness). Together, these aligned determinants shape implementation impact—particularly reach, adoption, acceptability, fidelity, sustainability, and equity—highlighting the need to treat racism as a core contextual factor in implementation planning and evaluation. Created in BioRender. Grant, T. (2026), https://BioRender.com/3uvcrcg (accessed September 21, 2026); License: Agreement number BH2A9M6KBJ; Source: BioRender.

## 3 Racism mediated adaptive responses

While medical mistrust can sometimes lead to harmful outcomes, it can also function as an empowering mechanism that drives the development of powerful resilience strategies in response to sustained exposure to racism and medical harm. Sisto defines resilience as “the ability to maintain the persistence of one's orientation towards existential purposes. It constitutes a transversal attitude that can be understood as the ability to overcome the difficulties experienced in the different areas of one's life with perseverance, as well as good awareness of oneself and one's own internal coherence by activating a personal growth project” (Sisto et al., 2019). Historically, racism has mobilized communities of color to mitigate its impact. From the Jim Crow era through the COVID-19 pandemic, communities of color have consistently found ways to survive in the face of profound adversity. In moments of crisis, many minoritized communities have shifted power away from formal government institutions toward local, community-rooted systems of care, leadership, and survival. These responses often produced results that were faster, more culturally grounded, and more equitable than official public health efforts. These resilience responses are not only survival strategies, but also expressions of power, autonomy, resilience, and collective care in the face of systemic neglect.

Within this context, mistrust of the healthcare system is not irrational; it is a learned response to repeated experiences of racism and exclusion. Thus, individuals experiencing mistrust in the healthcare system often turn to credible, culturally attuned individuals and organizations as a resilience strategy, seeking guidance outside formal care settings and relying instead on family, friends, community leaders, and local organizations as trusted messengers (24). A common resilience mechanism is the formation of community-based organizations (CBOs), which are nonprofit organizations that represent a community and aim to meet its specific needs (25). CBOs often employ members of the community they serve who have earned trust through their longstanding engagement or shared lived experience (24). Trusted messengers bring the authority and familiarity needed to achieve uptake and build confidence in health messages because they understand and are embedded within the communities they serve (26). During the COVID-19 pandemic, many marginalized communities used CBOs and trusted messengers to combat misinformation, encourage vaccine uptake, and provide highly effective outreach, education, and vaccination efforts (27, 28). CBOs and trusted messengers are examples of racism-mediated resilience, but other adaptive strategies include mutual aid and informal care networks, faith-based institutions, and grassroots advocacy. Collectively, these function as parallel public health infrastructures that shape how, when, and where communities engage with health information and services.

Resilience is not only a form of self-preservation, but also a mechanism of empowerment, enabling communities of color to safeguard themselves while reducing exposure to harm and the impact of longstanding disparities. It reflects the reality of surviving in often hostile environments and the need to build trust and engagement outside formal medical institutions. Although health disparities in minoritized communities have been well documented, less attention has been paid to the community-led responses that have grown out of generations of racism. For implementation science, these adaptive strategies are not side notes; they are essential community assets that need to be understood, respected, and actively partnered with if interventions are to be accepted, adapted, and sustained.

## 4 The racism, resilience response framework

Racism doubles as both a facilitator and a barrier to implementation: it produces health inequities, but it also forces individuals who experience racism to develop adaptive resilience mechanisms that can be leveraged to improve implementation success. Intervention environments play a major role in shaping outcomes, and for minority populations these environments are deeply influenced by racism. Most implementation science frameworks do not account for contextual determinants such as power and racism. They are implemented generically across all populations with little regard for lived experience and the impact of these determinants (29). Moreover, implementation frameworks are broad in scope and fail to address issues specific to minoritized groups, further exacerbating health inequities in already vulnerable populations (30, 31). If implementation science is to make meaningful progress toward health equity, it must explicitly address the needs of minoritized groups in the design and implementation of interventions (29). Racism must be explicitly embedded within implementation frameworks, and implementation strategies should include mechanisms that directly address health equity (2).

Implementation science is not lacking frameworks; what is lacking is an understanding of how to adapt and apply existing frameworks to address racial and ethnic inequities in healthcare (32). The Health Equity Implementation Framework (HEIF) was intentionally designed to identify factors related to uptake, healthcare disparities, and improved outcomes in marginalized communities (33, 34). It incorporates culturally relevant factors, the clinical encounter, and societal context, but racism remains largely implied rather than directly specified. Integrating racism and its adaptive responses into HEIF strengthens its capacity to account for lived experience and community-generated infrastructures that shape implementation uptake, acceptability, sustainability, and success.

Public Health 3.0 is a contemporary public health framework that emphasizes cross-sector collaboration to address the root causes of disease, such as income inequality, structural racism, and environmental hazards, to achieve health equity. It relies on partnerships among communities and public and private sectors to take public health “to the next level” through shared funding, services, governance, and collective action. It emphasizes the collection of timely and reliable data to deepen understanding of community needs, inform programming, and track the impact of new initiatives (35). Within this paradigm, Ellis's Community Resilience (CR) Model offers a Public Health 3.0-aligned approach specifically aimed at defining and measuring community resilience to assess equity, confront racism, and improve population health (36). Ellis defines CR as: “The sustained ability of community systems to prepare for, withstand, and recover from acute shocks while addressing and preventing the chronic adverse effects of structural racism, and a community's ability to cope, strive, and be supported through equitable access to buffers that address and relieve sources of chronic stress and acute adversity” (36).

Traditional implementation science approaches employ a “top-down” approach, emphasizing expertise from academics and researchers, while relegating community members and other vital stakeholders to more passive consultation roles (37, 38). A recent assessment of active implementation science grants found that only 3% included community engagement through a partnership model, which is considered the highest level of engagement (37, 39). However, participatory approaches that include multi-sector collaborative partnerships involve an iterative co-creation of knowledge and a vital deviation from the practice of forcing EBIs to work within harmful systems toward implementation efforts that transform systems to center equity (38, 40). Thus, combining the HEIF with the CR model presents a powerful tool for implementation science to not only promote health equity, but to shape systems through multi-sector partnerships designed for and by the communities in which interventions are implemented. Thus, the Racism–Resilience Response Framework (R3F) is proposed as a modified, equity-centered extension of the HEIF that utilizes the CR model to promote implementation success and advance health equity explicitly within historically marginalized communities (Figure 2). The R3F is intended for use across multiple intervention levels, ranging from individual-level clinical programs to broader community and public health settings.

**Figure 2:**
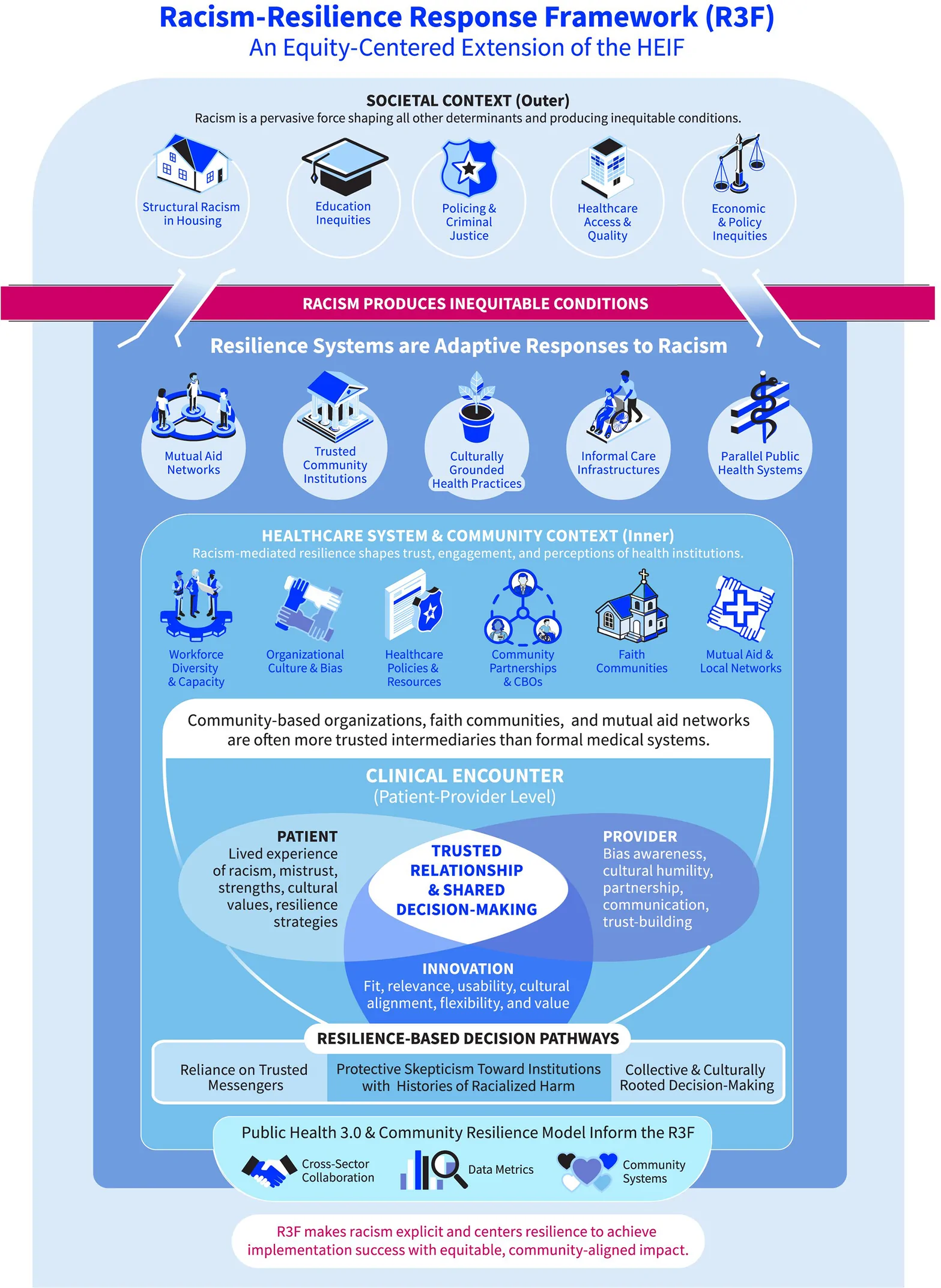
Racism–resilience response framework (R3F): an equity-centered extension of HEIF. The R3F builds on the HEIF, Public Health 3.0, and the Community Resilience model to explain implementation in minoritized communities. In the societal (outer) context, institutional racism in housing, schooling, policing, health access, and economic policy creates unequal contexts, and in response, communities establish resilience systems- mutual aid networks, trusted CBOs, culturally grounded health practices, informal support infrastructures- in what constitutes a parallel public health system. These factors shape the healthcare system and community context (inner), including workforce diversity and capacity, organizational culture and bias, healthcare policies and resources, and relationships with CBOs, religious groups, community organizations, and mutual aid networks. In the inner context, the clinical (patient-provider) encounter will be based on a trusted relationship and shared decision-making between patient and provider, mediated by culturally relevant, flexible, and user-friendly interventions. Patient and provider experiences are mediated by racism and resilience, where patients bring lived experiences of racism, mistrust, culture, strengths, and resilience strategies, and providers bring racial bias awareness, cultural humility, communication skills, and trust-building capacity. As a result of these influences, patients and providers rely on resilience-based decision-making pathways: trusted messengers, collective and culturally rooted decision-making, and skeptical optimism of institutional sources with legacies of racism. The bottom band depicts the influence of Public Health 3.0 and the Community Resilience model on R3F, emphasizing cross-sector governance, structural racism measures, and resilience metrics. Collectively, these elements position racism and resilience as multilevel implementation determinants and guide the design of strategies that leverage community infrastructures.

Under the outer context, the R3F details how housing, education, policing, and healthcare access-based structural racism drive the inequitable conditions HEIF identifies as barriers. These structural forces shape risk while also generating community resilience structures—including mutual aid networks, trusted community institutions, culturally grounded health practices, and informal care infrastructures. Incorporating these resilience systems into the HEIF positions them as structural determinants that influence feasibility, reach, and resource alignment during implementation.

Within the inner context, racism influences trust levels, healthcare system engagement, and perceptions of healthcare organizations. The R3F asserts that CBOs and informal mutual aid networks may already function as effective messengers; thus, they should be involved in formalizing clinical services that patients feel comfortable using and in implementing public health interventions in minoritized communities. Engagement with these organizations and networks not only enhances the reach of health interventions, but given that they already operate as trusted infrastructure, their formal involvement can improve intervention uptake, acceptability, and sustainability.

At the patient-provider level, the R3F highlights how historical and ongoing medical harm simultaneously creates heightened mistrust and adaptive, culturally grounded coping strategies that shape health-seeking behaviors. The R3F expands the clinical encounter in the HEIF to include resilience-based decision strategies, such as relying on trusted messengers to make decisions about their personal and community healthcare, engaging in collective and culturally informed decision-making processes, and expressing protective skepticism toward institutions and providers with histories of racialized harm.

At the societal level, R3F is distinctive in that it explicitly asserts that racism is an all-encompassing root determinant on which all other determinants rest, specifying a model of racism-mediated resilience in response to, and as a moderator of, health care infrastructure on behalf of minority individuals and groups. Integrating racism and racism-mediated adaptations into the HEIF enhances researchers' ability to reflect the actual experiences of minoritized groups within inequitable systems, and it helps identify culturally appropriate, community-tested interventions to increase uptake and improve overall implementation. Through this integration, the R3F strengthens the HEIF's ability to influence the parameters of equity-focused implementation by more accurately accounting for how minoritized individuals navigate inequitable systems and identifies methods already used to resist and persist against racism when developing or implementing programs and policies. Consequently, the R3F provides an implementation framework that supports the adoption of innovation through a community-driven approach that promotes equitable outcomes.

While many examples discussed here are rooted in the experiences of Black communities, it is intended to be useful when applied to other racialized/ethnic groups. The ways racism manifests, the types of community resilience available, and the policy context will change, and application of the R3F requires sensitive consideration of local histories, power dynamics, and community priorities. Even within an ethnic or racial group, it is important to remember that not all people are alike. There is no “one size fits all” to apply to any community, as each community within a particular minoritized group has different resource systems and life experiences that are important to note when using the framework. Thus, the R3F is not designed to offer a set menu of interventions; rather, it provides an architecture to work with a community to understand their contexts in relation to racism and community resilience, and to co-develop implementable strategies. Consequently, operationalizing the R3F requires researchers to reconceptualize implementation as a collaborative redesign of systems rather than a unidirectional effort to “fit” evidence-based interventions into existing structures. Moreover, implementation is a cyclical, iterative process that requires continuous assessment and adaptation in response to emerging data. The proposed R3F process includes built-in feedback loops to support ongoing evaluation, refinement, and improvement over time (Figure 3).

**Figure 3:**
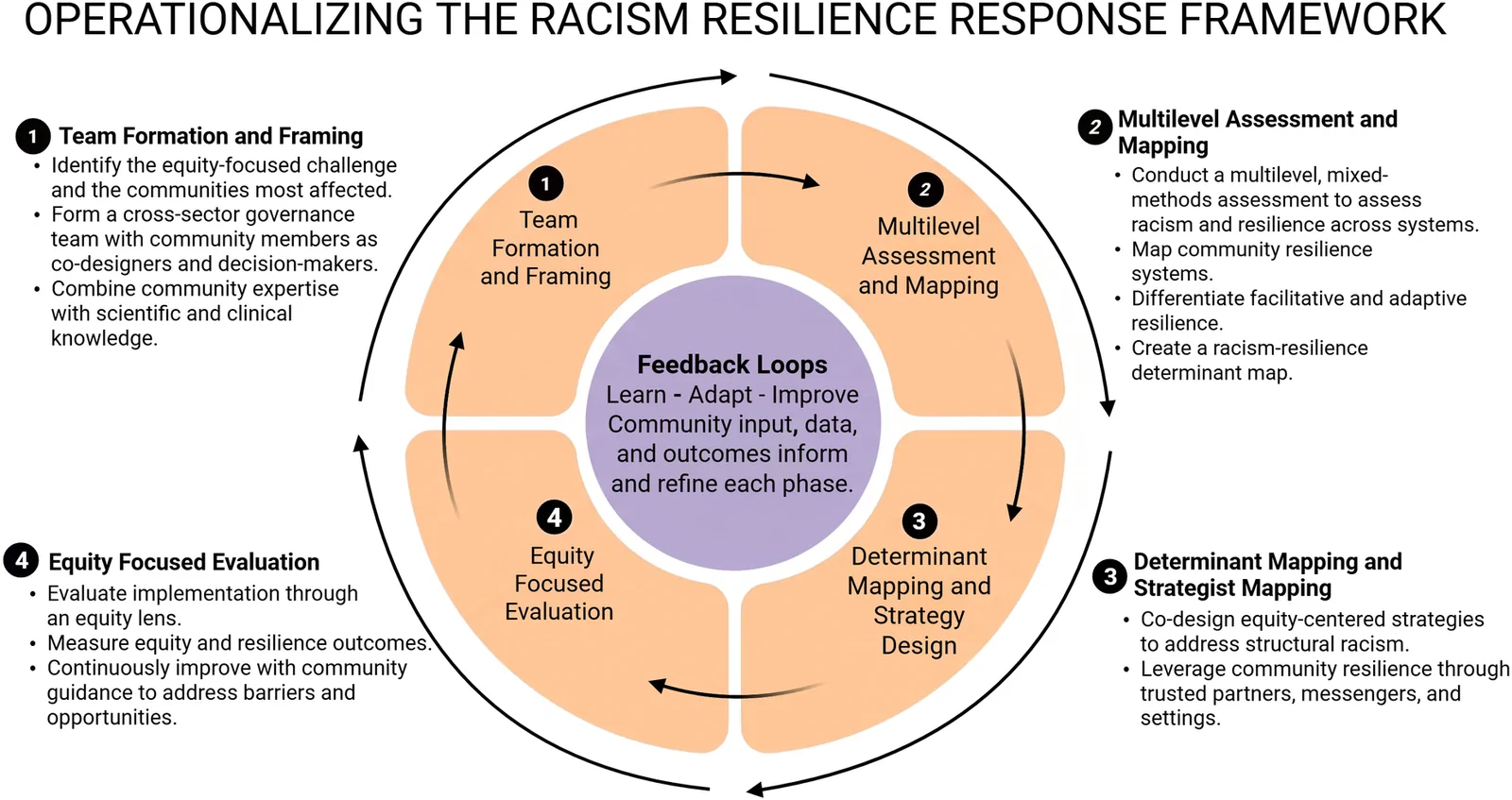
(41). Operationalizing the racism resilience response framework (R3F). The R3F provides a four-step, equity-centered approach for integrating considerations of structural racism and community resilience into implementation research and practice. The framework begins with team formation and framing, followed by multilevel assessment and mapping of racism-related barriers and resilience assets, determinant mapping and strategy design to address structural inequities and leverage community strengths, and equity-focused evaluation of implementation, equity, and resilience outcomes. The process is iterative and guided by community partnership, shared decision-making, and continuous adaptation to advance health equity. Created in BioRender. Grant, T. (2026), https://BioRender.com/ee636bf (accessed September 21, 2026); License: Agreement number UE2A9M74DT; Source: BioRender.

### 4.1 Step 1. Team formation and framing

The process begins by clearly defining the EBI and identifying the specific racialized communities that bear its greatest burden. As suggested by Public Health 3.0's focus on multisector coalitions, the next step involves assembling a multisector governance team that brings together implementation scientists, health services researchers, clinicians, and health system leaders, and representatives of community-based networks, which would ideally include community and faith-based leaders, mutual aid groups, and CBO leaders. The implementation team works with these community stakeholders as co-designers and co-decision-makers to identify priorities, interpret the evidence, and determine its use. This governance structure bridges lived experience from community members and scientific and clinical expertise, providing the context needed to produce a desirable and actionable strategy and intervention (38). The R3F requires the researcher to shift from asking how to make an EBI work “for” a community to asking how to reshape both the intervention and the systems around it so they advance equity in the face of racism-structured contexts.

### 4.2 Step 2. Multilevel assessment and mapping

Guided by this team, the next phases involve a multi-level, mixed-methods assessment to characterize the formal and informal community-generated health infrastructures into which the intervention will be introduced. On the structural level, information on historic redlining, segregation, school resources, access to transportation, housing code enforcement or zoning, and insurance or healthcare access is collected through geospatial analyses, policy review, and census indicators. At the inner, organizational level, interviews, surveys, and focus groups with organization staff, leadership, and community partners are used to explore resource allocation, leadership support, organizational culture and policies, operational procedures (e.g., referral pathways and language access), and existing partnerships with CBOs and faith groups. At the patient level, qualitative data are collected from patients, caregivers, and clinicians and used to document experiences of bias and discrimination, levels of trust and medical mistrust, and decision-making related to bias and racism. Where possible, these data are complemented by validated measures of medical mistrust, perceived discrimination, and patient-reported experience. Finally, at the societal level, investigators examine national and state policies, media coverage, and broader sociopolitical forces that shape local narratives about race, health, and institutions. All data collection should foreground the community-based, informal resilience systems, including mutual aid networks, faith institutions, culturally grounded healing practices, informal caregiving structures, and other community-based infrastructures. After identifying these systems, researchers examine how they currently interface with or substitute for formal healthcare.

In line with the CR Model's call to measure structural racism and community resilience, the resulting data are synthesized into a map showing how racism and resilience influence implementation. The map shows how racism impacts outcomes, for example, through transportation or insurance barriers or mistrust due to past discriminatory care. The map will also highlight how resilience systems support or shape engagement, such as when community-based organizations serve as trusted sources of health information or when faith leaders and other trusted messengers influence decisions about vaccines and treatment. Finally, it distinguishes between resilience mechanisms that facilitate implementation success, such as trusted messengers enhancing uptake, and those that arise as adaptive responses to structural barriers, such as reliance on alternative sources of care when hospitals and clinics are perceived as unsafe or untrustworthy.

### 4.3 Step 3. Determinant mapping and strategy design

Using this determinant map, the governance team co-designs equity-centered implementation strategies that intentionally interrupt and respond to structural racism. Examples include modifying eligibility criteria, decentralizing service delivery, or modifying accountability indicators. The team intentionally leverages resilience systems by formalizing partnerships with CBOs, building the capacity of trusted messengers to deliver interventions or serve as navigators, and integrating services in faith communities and other trusted community venues. Importantly, the team focuses on avoiding the replication of inequities by supporting and compensating under-resourced community organizations rather than further burdening them with additional responsibilities without sufficient support.

### 4.4 Step 4. Equity-focused evaluation

Evaluation within the R3F is structured with an equity and resilience lens. Traditional implementation outcome measures are measured and disaggregated by race/ethnicity, neighborhood, and other relevant equity indicators. Additionally, other equity-focused outcomes are measured, including changes in racial and ethnic gaps in intervention uptake and clinical outcomes, shifts in trust and perceived discrimination among minoritized individuals and communities, and organizational changes in policy, resource allocation, and partnerships with community entities. Engagement with resilience systems is monitored through engagement with CBOs and trusted messengers, and community-defined indicators of success are measured and evaluated for attainment and translation of outcomes. Qualitative methods are used to examine alignment with community priorities, perceptions of anti-racism, and how implementation strategies interact with and transform existing resilience structures.

Finally, because implementation in racialized contexts occurs in dynamic settings and is shaped by multilevel systems, the R3F moves beyond traditional linear approaches and recognizes that implementation requires an iterative approach. Community governance structures, which include advisory boards with genuine decision-making authority, guide implementation and provide ongoing oversight and interpretation of quantitative and qualitative data. These governance structures guide adaptations to implementation strategies in response to newly identified racism related barriers and resilience-related opportunities, and they support continuous refinement of both the intervention and the framework itself. The R3F treats implementation not as a linear, one-directional process of fitting interventions into existing settings, but as an iterative process where both racism and resilience interact across multiple levels to shape how interventions are received, adapted, and sustained. Thus, the R3F provides researchers with an actionable roadmap for integrating racism and resilience in all phases of intervention development, delivery, and evaluation, making equity not an aspirational goal but a core operational tenet of implementation design.

## 5 Discussion

This work advances equity-centered implementation science by framing racism and community resilience as multi-level determinants of implementation and by offering the Racism-Resilience Response Framework as a practical way to translate these concepts into implementation planning. Building on the Health Equity Implementation Framework and the Community Resilience model, the R3F moves beyond traditional race-neutral applications of existing frameworks. It reframes implementation efforts to design, deliver, and evaluate interventions that are community-anchored, explicitly anti-racist, and responsive to lived experience. The framework foregrounds the role of racism in shaping implementation conditions while also recognizing community resilience as a source of knowledge, infrastructure, and adaptive capacity that can support more equitable uptake and sustainability.

A central contribution of the R3F is its shift from the traditional linear view of implementation to a more dynamic, systems-oriented perspective. The R3F asserts that interventions do not enter static settings; rather, they are introduced into systems heavily shaped by racism, institutional disinvestment, community mistrust, organizational constraints, and community resilience. Each of these interacts at multiple levels, in relation to the others, and over time, creating feedback loops that affect the success of intervention efforts. By underscoring systems, feedback, and iteration, the R3F advances current equity-centered implementation by arguing that implementation in minoritized communities requires ongoing reassessment, partnership, and adaptation rather than a one-time deployment. Consequently, the R3F advances current knowledge by offering a more realistic description of how equity-focused implementation should unfold in complex, inequitable contexts.

Additionally, the R3F has important implications for implementation practice. The framework foregrounds the lived experience and impact of racism on minoritized individuals and approaches implementation in community-centered ways intentionally designed to promote trust and facilitate more successful outcomes. Its participatory nature draws directly from community expertise to support the development of culturally tailored, community-driven interventions, and its engagement with community stakeholders and multilevel data collection on racism and resilience moves implementation efforts beyond passive consultation or performative action toward success (Figure 4).

**Figure 4:**
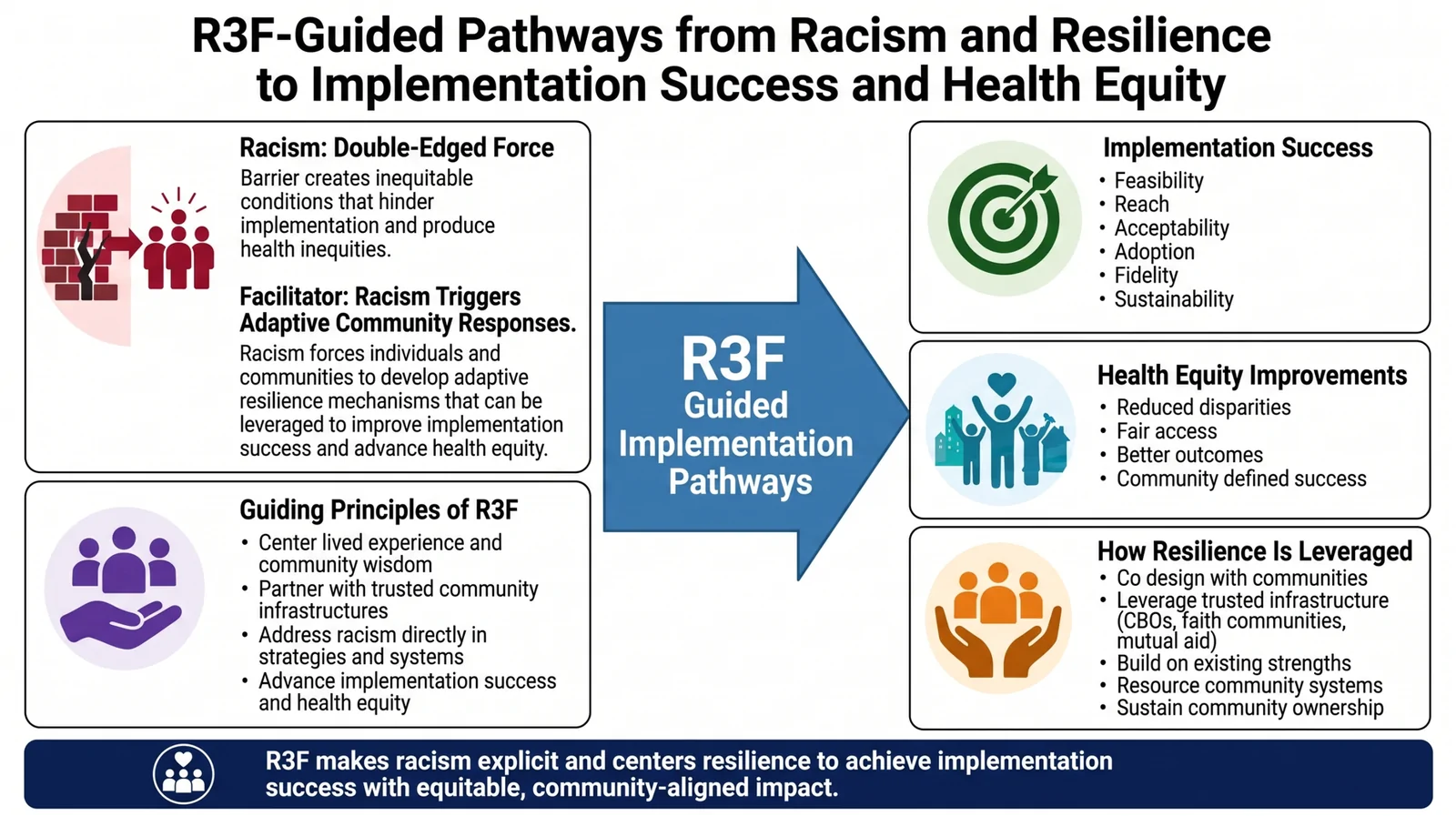
R3F-guided pathways from racism and resilience to implementation success and health equity (42)**.** The Racism–Resilience Response Framework (R3F) theorizes racism as a double-edged force that creates inequitable conditions and health inequities while forcing communities to develop adaptive resilience responses. By centering lived experience, partnering with trusted community infrastructures, and addressing racism directly, the R3F leverages resilience_based decision pathways to shape implementation pathways. These pathways lead to more frequent implementation success and health equity improvements by intentionally leveraging resilience through cross-sector partnerships that co-design interventions, use trusted community infrastructures and resource community systems, and sustain community ownership. Created in BioRender. Grant, T. (2026), https://BioRender.com/8rbr0lw (accessed July 31, 2026); License: Agreement number EH2A0YPERX; Source: BioRender.

Evidence from multisector community partnerships underscores the importance of this approach. During the COVID-19 pandemic, community-based participatory research methodologies led to community-reported improvements in access to testing and information, the implementation of diverse communication strategies to support risk mitigation, and informed equity-focused federal policy recommendations in African American, Native American, and Latinx communities (43). Cultural and communication mismatches can pose substantial barriers to implementation and generate resistance among diverse populations. However, one recent study found that multisector partnerships leveraged community-level data to address language, transportation, and literacy barriers, leading to the successful vaccination of 5,000 community members (44). The authors of the study concluded that community partnerships that address SDoH can drive reciprocal improvements in community resilience and advance health equity (44). Long-term estimates also suggest that sustained multisector partnerships launched through these collaborations could prevent 880 premature deaths and save approximately $125.7 million in medical costs over 20 years (45). Collectively, these studies reinforce the long-term value of coordinated, cross-sector partnerships in addressing drivers of health inequities and highlight the type of community-anchored, iterative implementation work that the R3F is designed to support.

Applying an antiracism lens in implementation research helps identify racism as a root cause of health inequities and prompts researchers and practitioners to critically examine how racism embedded in policies and systems differentially affects minoritized groups (3). The R3F addresses this by specifying how structural racism and resilience systems influence implementation processes and providing a step-wise process for incorporating these determinants into planning, strategy design, and evaluation. By making racism and resilience explicit multilevel determinants rather than background conditions, the R3F challenges researchers, health systems, and policymakers to redesign implementation processes so that equity is not an afterthought but a central organizing principle.

From a conceptual perspective, the R3F can be viewed as implementation science's embodiment of Public Health 3.0 and the Community Resilience model, offering a multilevel approach to implementation planning in clinical, community, and population health settings. By situating multi-sector governance, structural measures of racism, and attention to community resilience systems into implementation planning and evaluation, the R3F operationalizes these in both clinical and community settings. However, the R3F is still a concept and a methodology for implementing interventions in real-world settings that require empirical testing across various contexts.

There are a number of challenges to implementing the R3F. It will take considerable time, resources, and expertise that many implementation teams do not currently possess. Most significantly is identifying appropriate ways to measure structural racism and resilience, and the challenge of understanding how these mechanisms operate and interact across the levels and settings described in the R3F. Although there are validated measures of related concepts such as medical mistrust, mistrust is conceptualized and operationalized in various ways (46) and implementation teams will need to select measures that best fit their implementation context and desired outcomes. Additionally, successful implementation depends on fostering authentic, trusting community partnerships, and developing trust takes time, particularly in environments where communities and individuals have been previously harmed or have experienced performative engagement. Moreover, the multilevel assessments, community engagement, and ongoing measurements inherent to the R3F require considerable time, staffing, funding, and expertise that may not be readily available. Finally, implementation teams should avoid overburdening community partners, especially when their resources are limited. I argue that these challenges do not diminish the framework's value. They emphasize why an approach like the R3F is needed to guide more thoughtful, responsive, and equitable implementation efforts over time.

Continued study should prospectively apply the R3F across multiple implementation projects, begin to evaluate how R3F-guided strategies compare with conventional methods on equity-related implementation outcomes, and develop practical tools such as measures, checklists, and decision aids to support its use. Although this article presents the R3F as a conceptual framework and empirical testing is still needed, it nonetheless offers a practical roadmap to guide future research and practice toward equity-centered, community-aligned impact. Its broader contribution is not only that it adapts existing frameworks, but that it advances implementation science toward a more systems-aware, iterative, and equity-centered understanding of how interventions can succeed in historically marginalized communities.

## Data Availability

The original contributions presented in the study are included in the article, further inquiries can be directed to the corresponding author.
